# Supplementary material for: Interaction of an Acinetobacter baumannii’s Membrane Protein with Human Fibronectin to Evade Immune Response
Source: Chemistry. 2025 Jul 25;31(45):e00874. doi: 10.1002/chem.202500874 (PMC12351444; doi:10.1002/chem.202500874)
Supplement: Supplementary file 1 — Supporting Information [file CHEM-31-e00874-s001.pdf]

Supplementary Information for

Interaction of an *Acinetobacter baumannii*'s Membrane Protein with Human Fibronectin to Evade Immune Response

Laurine Vasseur [a], *Florent Barbault* [a*], *Antonio Monari* [a*].

a) Université Paris Cité and CNRS, ITODYS, F-75006 Paris, France.

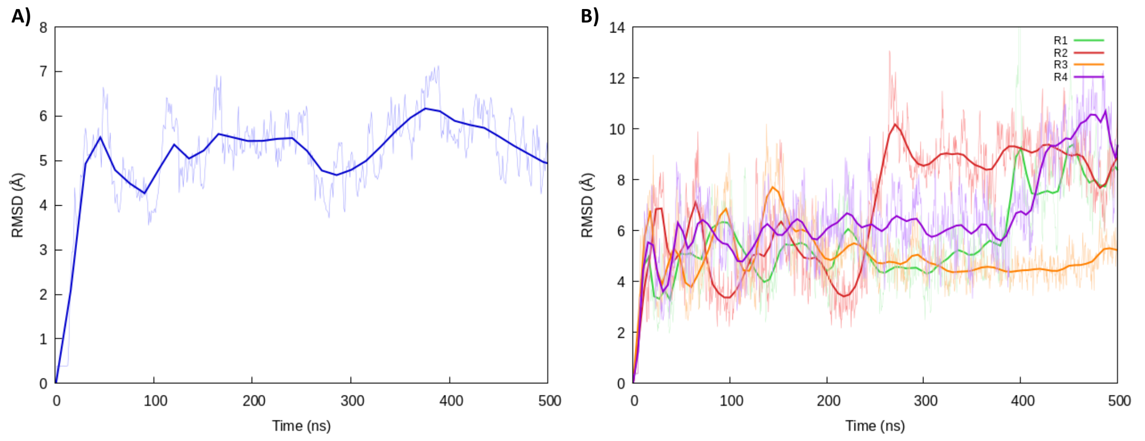

Figure S1. A) RMSD of FN averaged over the replicas. B) Individual RMSD time evolution.

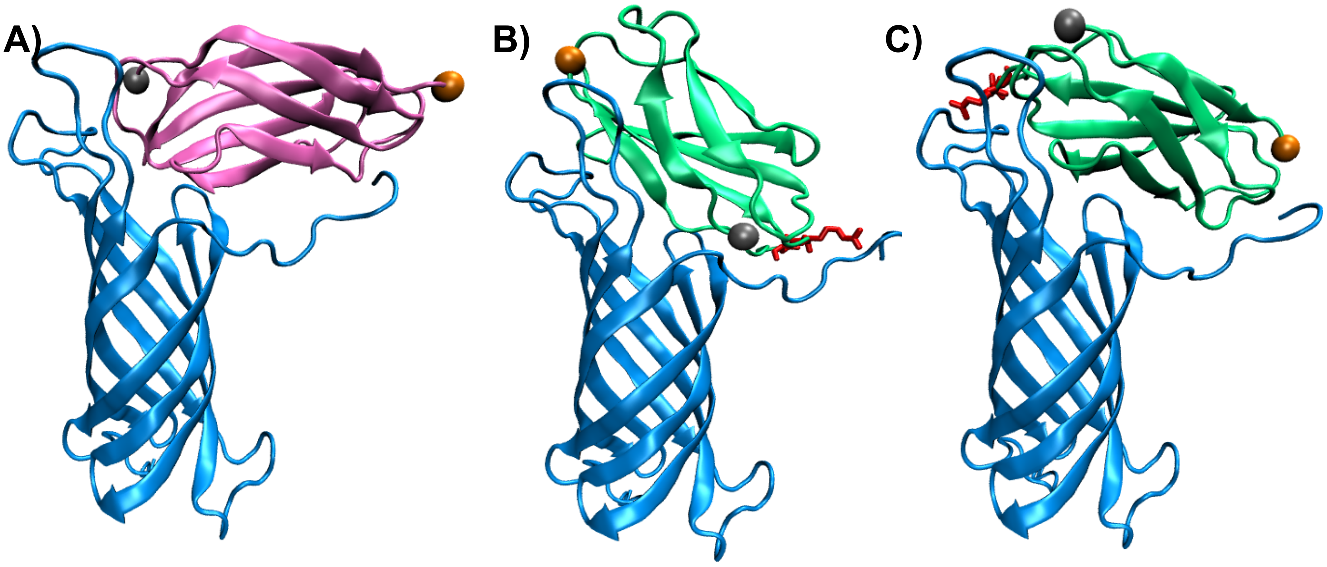

Figure S2. Poses of FN/OmpA complex obtained after docking. A) nonRGD (pink), B) RGD_1 (green), C) RGD_2 (green). OmpA is always represented in blue.

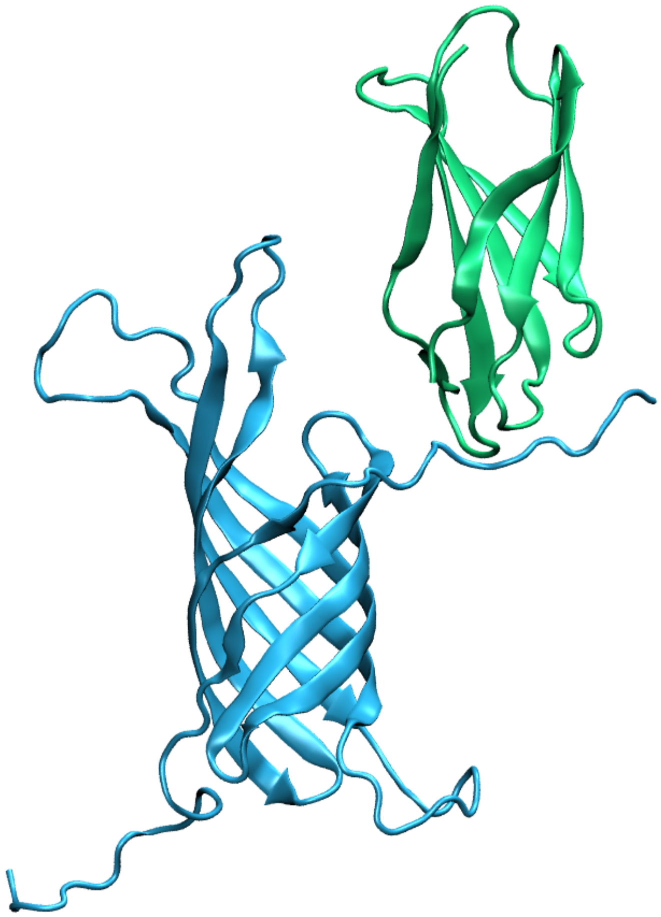

Figure S3. Structure of the unphysical binding between OmpA and RGD_1. This structure has been discarded from further analysis since the structure is not compatible with an extended FN unit. OmpA is represented in blue and RGD_1 in green.

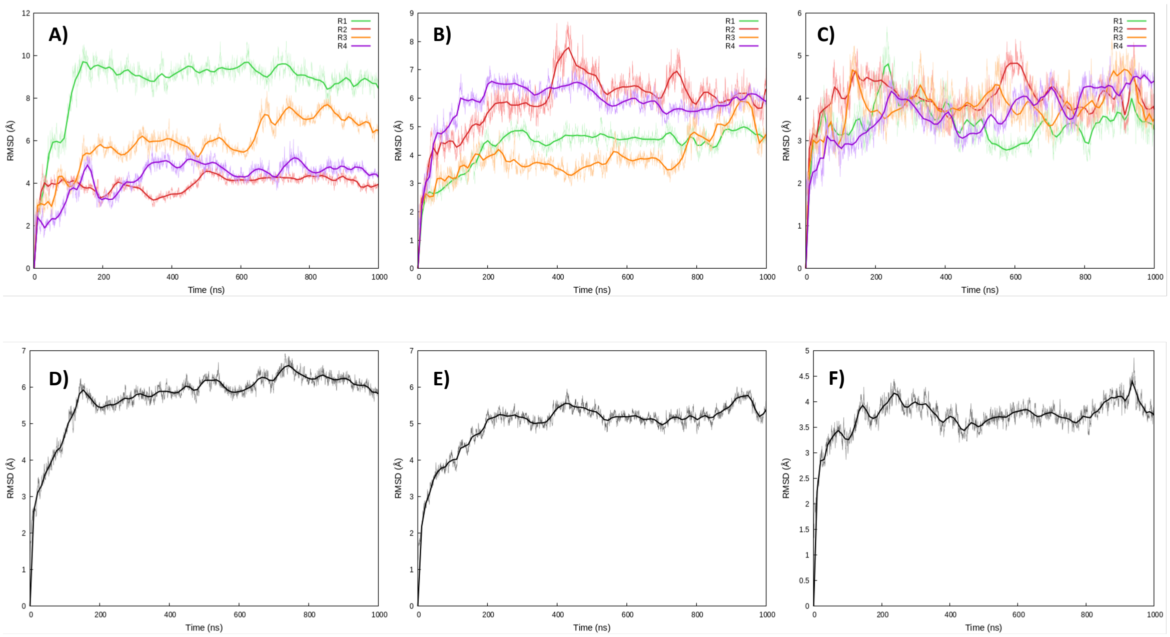

Figure S4. Time evolution of the protein RMSD for ABM interacting with one FN. Pose RGD_1 individual replicas (A) and the average (D). Pose RGD_2 individual replicas (B) and the average (E). Pose non RGD individual replicas (C) and the average (F).

RGD_1	R1	R2	R3	R4	Total
**D42 - R367**	0.81	99.01	0.1	20.1	**39.7**
**D1 - R352**	2.15	0.04	17.3	74.6	**30.6**
**E9 - R280**	0	0.08	0.2	91.4	**30.6**
**E134 - K360**	0.22	0	22.0	36.8	**19.6**
**D40 - K360**	6.15	6.07	40.4	4.1	**16.9**
**D13 - K360**	2.52	5.59	3.0	23.0	**10.5**
**D12 - R280**	1.15	1.47	0.0	22.9	**8.1**

Table S1. Percentage of presence of electrostatic interactions between OmpA and RGD_1, over the 1µs of simulation time. Gray cases report the values for the unphysical replica not considered (see Figure S2). The color of cases depending on the persistence of the interaction, blue: <7%; green: 7%≤x<20%; yellow: 20%≤x<40%; orange: 40%≤x<60% and red: ≥ 60%.

RGD_2	R1	R2	R3	R4	Total
**E134 - K328**	17.7	13.7	88.7	31.8	**38.0**
**E134 - R304**	17.8	0.0	0.0	72.6	**22.6**
**D1 - K328**	0.0	0.0	0.0	80.7	**20.2**
**D40 - K328**	4.0	17.3	0.0	42.0	**15.8**
**E101 - K328**	0.0	49.5	2.7	0.4	**13.2**
**E9 - K328**	40.7	0.0	0.1	5.7	**11.6**
**E101 - R352**	0.0	0.0	0.0	44.2	**11.0**
**D42 - K328**	0.1	21.2	0.0	22.6	**11.0**
**D13 - K328**	5.2	1.2	0.7	28.5	**8.9**

Table S2. Percentage of presence of electrostatic interactions between OmpA and RGD_2, over the 1µs of simulation time. The color of cases depending on the persistence of the interaction, blue: <7%; green: 7%≤x<20%; yellow: 20%≤x<40%; orange: 40%≤x<60% and red: ≥ 60%.

nonRGD	R1	R2	R3	R4	Total
**E135 - R262**	51.4	7.0	67.2	5.5	**32.8**
**E135 - R233**	27.8	0.6	12.2	81.3	**30.5**
**D133 - R262**	51.2	8.1	45.8	6.4	**27.9**
**E9 - R238**	19.2	6.1	25.0	44.5	**23.7**
**D12 - R238**	24.0	2.0	23.9	4.5	**13.6**
**D1 - R228**	0.0	0.0	0.1	41.2	**10.3**
**E135 - R217**	19.0	13.8	0.6	0.2	**8.4**

Table S3. Percentage of presence of electrostatic interactions between OmpA and nonRGD, over the 1μs of simulation time. The color of cases depending on the persistence of the interaction, blue: <7%; green: 7%≤x<20%; yellow: 20%≤x<40%; orange: 40%≤x<60% and red: ≥ 60%.

RGD_1	R1	R2	R3	R4	Total
**E9 - D277**	37.7	40.3	28.76	62.84	**43.98**
**E9 - R280**	0	0	0	104.84	**34.95**
**D42 - R367**	0.3	58.2	0	22.56	**26.93**
**D40 - S363**	0.0	65.5	0	0	**21.82**
**Y42 - N365**	0.1	46.5	0	0	**15.50**
**Y130 - S355**	0.0	37.7	0	0	**12.55**

Table S4. Percentage of presence of hydrogen bonds between OmpA and RGD_1, over the 1µs of simulation time. Gray cases report the values for the unphysical replica not considered (see Figure S2). The color of cases depending on the persistence of the interaction, blue: <7%; green: 7%≤x<20%; yellow: 20%≤x<40%; orange: 40%≤x<60% and red: ≥ 60%.

RGD_2	R1	R2	R3	R4	Total
**E134 - R304**	6.0	0.0	0.0	56.5	**15.6**
**E134 - K328**	2.2	0.0	39.8	0.0	**10.5**
**D1 - T288**	34.6	0.0	0.0	0.0	**8.7**
**D1 - K328**	0.0	0.0	0.0	32.9	**8.2**

Table S5. Percentage of presence of hydrogen bonds between OmpA and RGD_2, over the 1µs of simulation time. The color of cases depending on the persistence of the interaction, blue: $<7\%$; green: $7\% \leq x < 20\%$; yellow: $20\% \leq x < 40\%$; orange: $40\% \leq x < 60\%$ and red: $\geq 60\%$.

nonRGD	R1	R2	R3	R4	Total
**E134 - T214**	28.7	9.2	35.9	9.8	**20.9**
**E134 - R233**	9.4	0.0	5.2	48.6	**15.8**
**E135 - R233**	15.9	33.2	7.7	4.5	**15.3**
**D1 - D232**	0.1	0.0	44.4	12.6	**14.3**
**E134 - G261**	20.5	5.6	1.8	27.0	**13.7**
**G2 - D232**	0.0	0.0	41.8	5.2	**11.8**
**T5 - T242**	0.1	0.0	40.1	6.3	**11.6**
**D133 - R262**	26.9	2.0	14.3	2.7	**11.5**
**E135 - R217**	4.1	37.4	0.0	0.0	**10.4**
**E101 - T212**	0.2	0.0	32.4	6.3	**9.7**
**Y50 - N260**	24.7	3.3	4.7	0.0	**8.2**

Table S6. Percentage of presence of hydrogen bonds between OmpA and nonRGD, over the 1μs of simulation time. The color of cases depending on the persistence of the interaction, blue: <7%; green: 7%≤x<20%; yellow: 20%≤x<40%; orange: 40%≤x<60% and red: ≥ 60%.

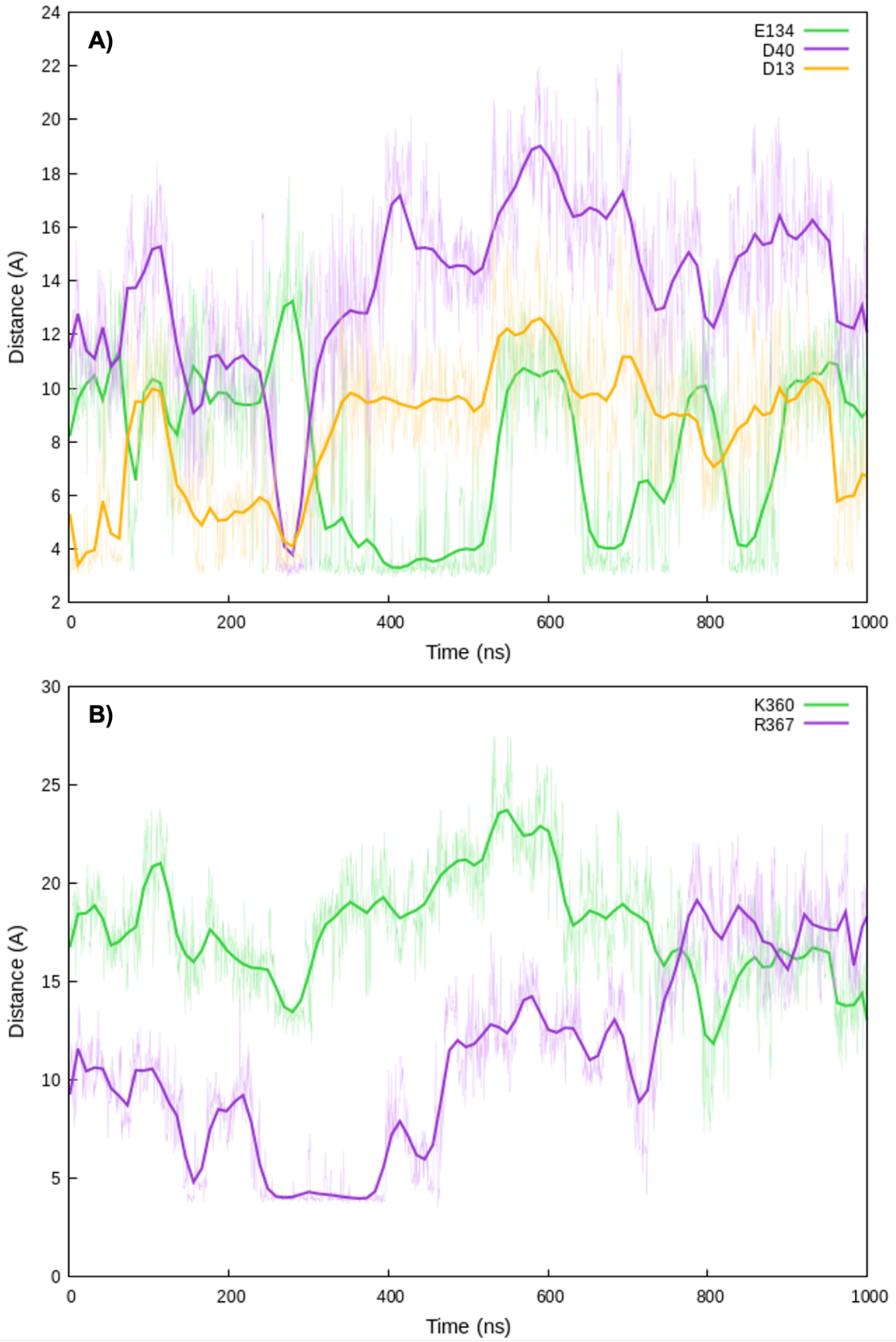

Figure S5. Timeseries of the distance between the residue K360 A) and residue D42 B) and all the closest residues for the RGD_1 complex.

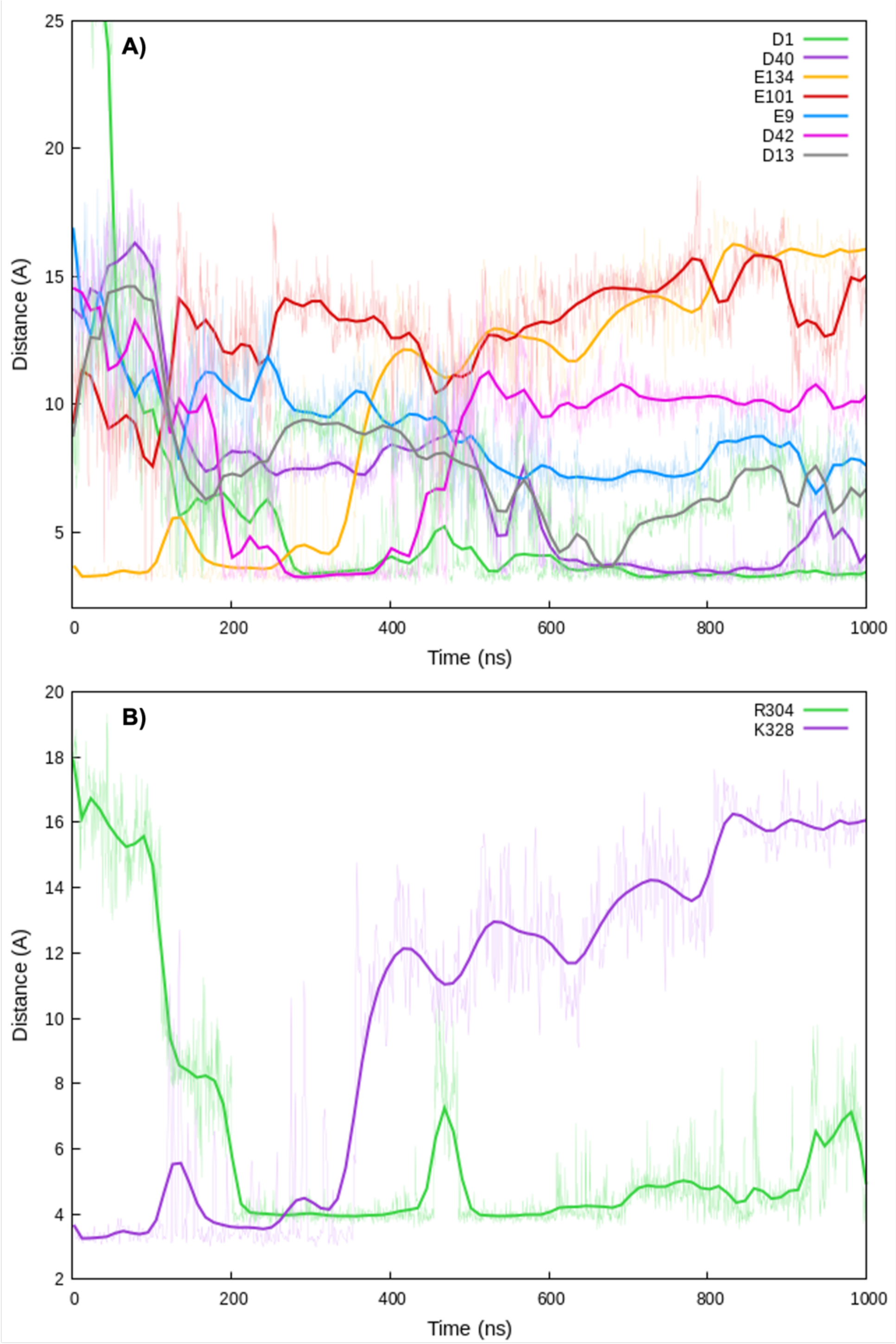

Figure S6. Timeseries of the distance between the residue K328 A) and residue E134 B) and all the closest residues for the RGD_2 complex.

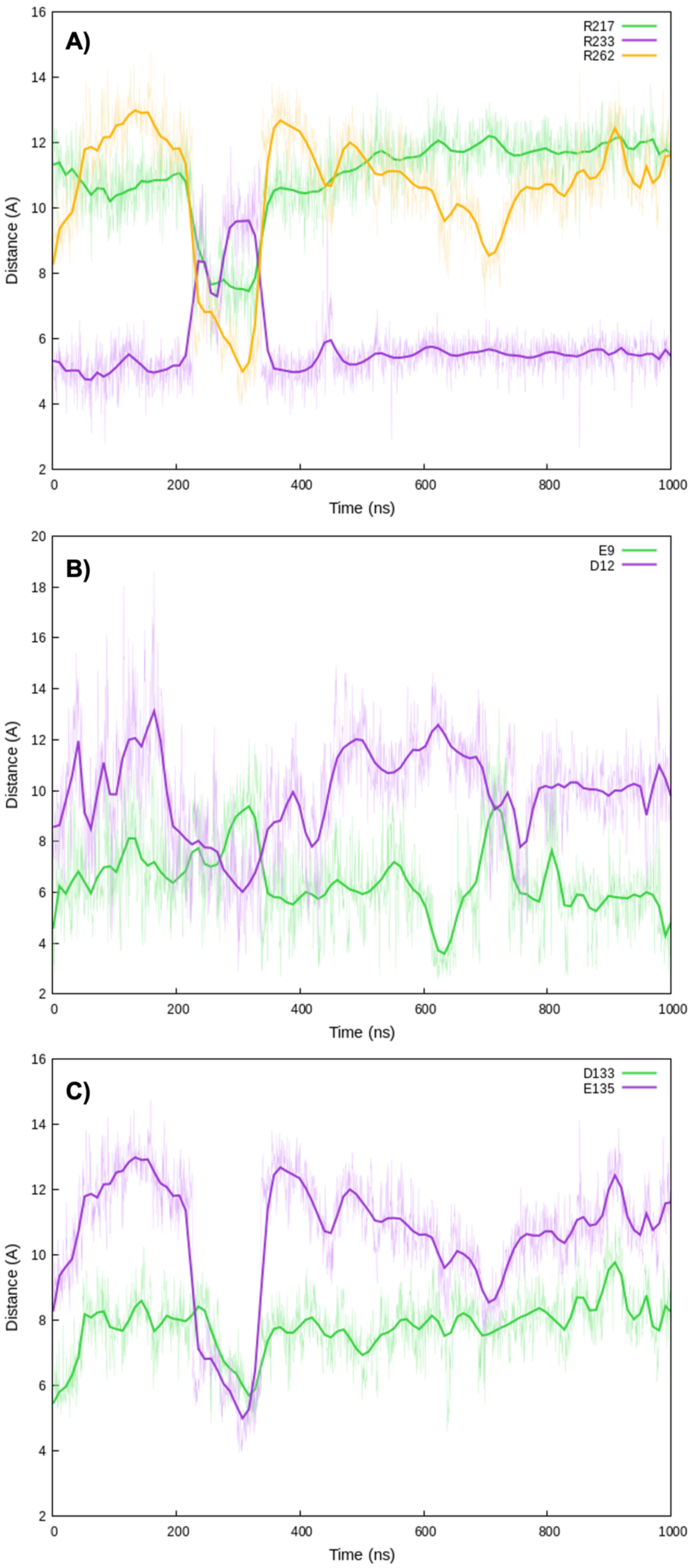

Figure S7. Timeseries of the distance between the residue R262 A), residue R262 B), and residue R238 C) and all the closest residues for the non-RGD complex.

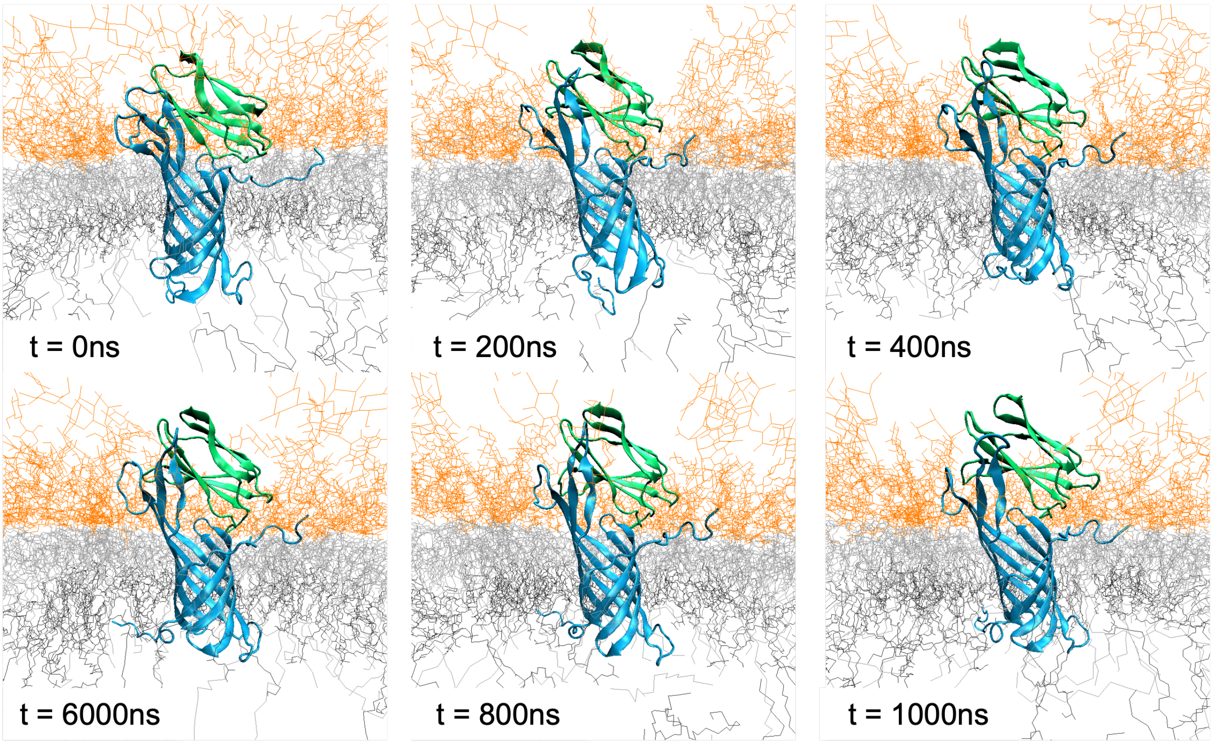

Figure S8. Snapshots along the MD simulation illustrating the OmpA/FN complex

RGD1

ABM	Fibronectin	ΔE_{elec}			ΔE_{vdw}			$\Delta E_{non\text{-}bonded}$		
**D134**	**K360**	-30.9	±	6.3	+0.3	±	1.1	-30.7	±	6.2
**D13**	**K360**	-9.6	±	3.9	+0.0	±	0.6	-9.7	±	3.9
**D40**	**K360**	-10.0	±	4.7	+0.0	±	0.9	-9.9	±	4.6
**D42**	**K360**	-5.9	±	4.1	+0.0	±	0.6	-5.8	±	4.1
**D42**	**K367**	-26.9	±	6.2	+0.5	±	1.1	-26.4	±	6.1

RGD2

ABM	Fibronectin	ΔE_{elec}			ΔE_{vdw}			$\Delta E_{non\text{-}bonded}$		
**D12**	**K328**	-8.8	±	3.6	+0.0	±	0.3	-8.9	±	3.6
**D13**	**K328**	-8.9	±	3.8	+0.0	±	0.3	-8.9	±	3.8
**D1**	**K328**	-15.8	±	5.3	+0.4	±	1.0	-15.3	±	5.3
**D40**	**K328**	-11.8	±	4.9	+0.0	±	0.7	-11.8	±	4.9
**D42**	**K328**	-0.1	±	2.0	-0.1	±	0.4	-0.2	±	2.0
**E101**	**K328**	-23.7	±	6.1	+0.3	±	1.1	-23.4	±	6.1
**E134**	**K328**	-36.9	±	6.6	+0.4	±	1.1	-36.4	±	6.5
**E134**	**R304**	-16.9	±	5.3	-0.1	±	0.9	-17.0	±	5.3
**E135**	**K328**	-0.2	±	0.8	+0.0	±	0.1	-0.2	±	0.8
**E9**	**K328**	-19.1	±	5.4	+0.1	±	0.9	-18.9	±	5.3

Non-RGD

ABM	Fibronectin	ΔE_{elec}			ΔE_{vdw}			$\Delta E_{non\text{-}bonded}$		
**D12**	**R238**	-14.0	±	4.3	-0.2	±	0.9	-14.2	±	4.3
**D133**	**R262**	-36.5	±	4.6	-1.4	±	1.2	-37.9	±	4.7
**D40**	**R238**	-6.5	±	3.8	-0.2	±	0.8	-6.7	±	3.8
**E135**	**R217**	-26.9	±	5.2	-0.3	±	1.0	-27.2	±	5.2
**E135**	**R233**	-43.1	±	4.1	-0.9	±	1.2	-43.9	±	4.0
**E135**	**R262**	-19.7	±	3.8	-0.4	±	0.6	-20.1	±	3.8
**E9**	**R238**	-29.7	±	5.4	-0.3	±	1.2	-29.9	±	5.3
**D12**	**R238**	-14.0	±	4.3	-0.2	±	0.9	-14.2	±	4.3
**D133**	**R262**	-36.5	±	4.6	-1.4	±	1.2	-37.9	±	4.7
**D40**	**R238**	-6.5	±	3.8	-0.2	±	0.8	-6.7	±	3.8

Table S7. Main residue-pair interaction energies averaged over the MD simulations.

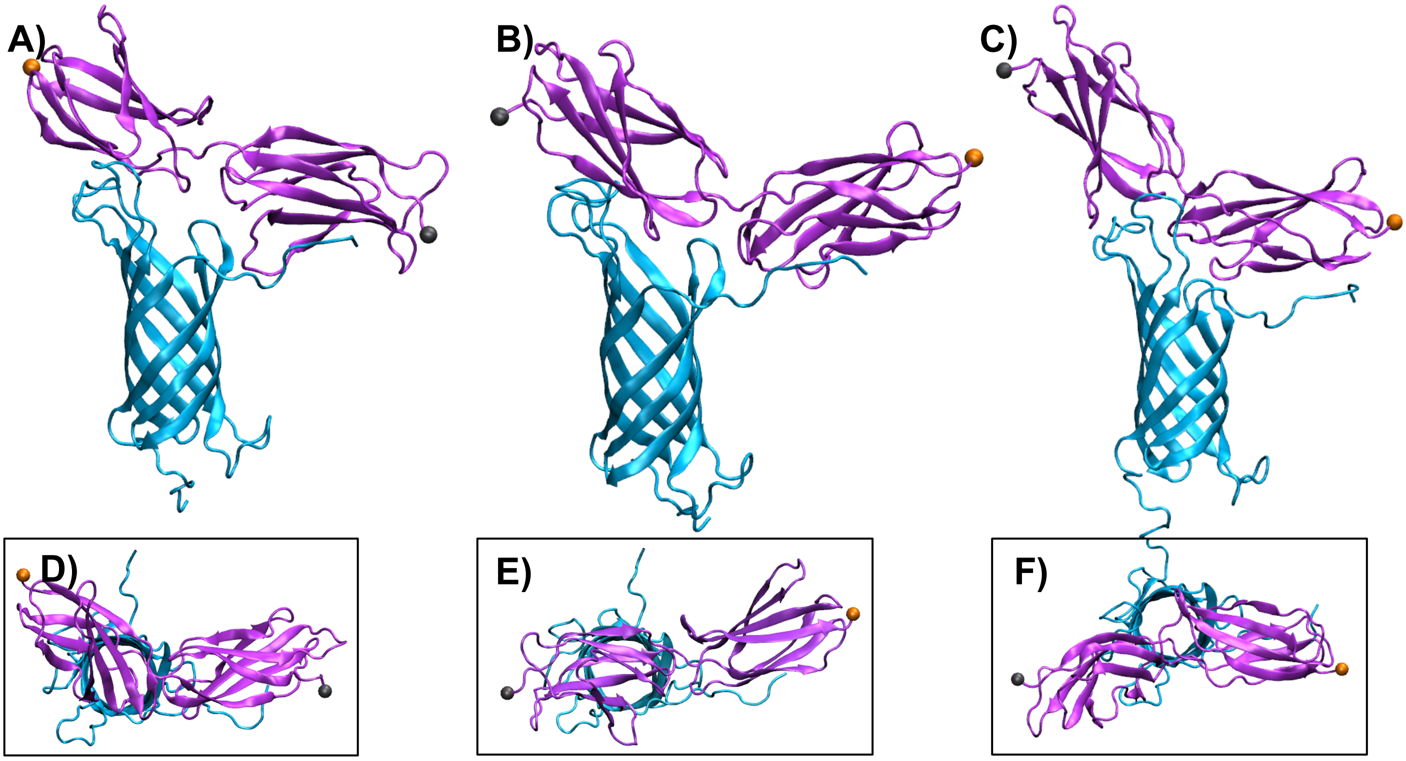

Figure S9. Docking poses for the complex between OmpA (blue) and two non-RGD beads of FN (purple). D), E) and F) are the top view of A), B) and C) respectively.

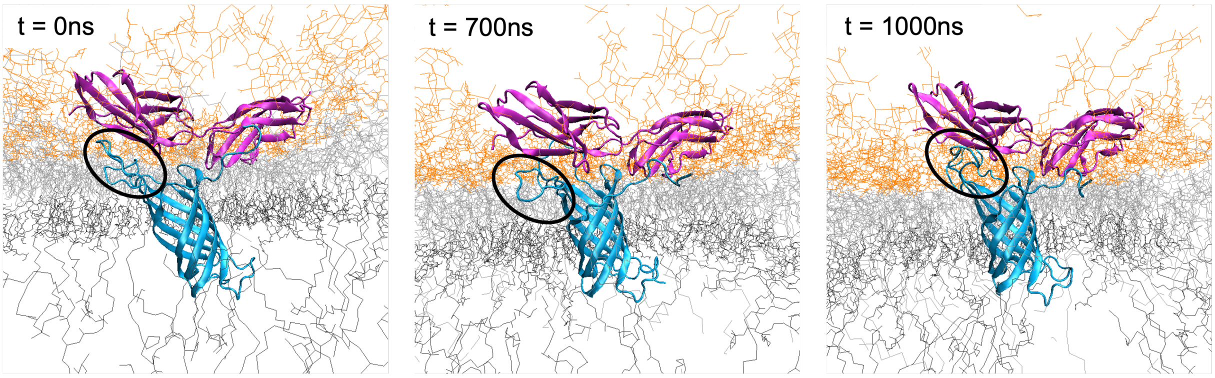

Figure S10. Snapshots showing the reorientation of the G39-K51 loop (evidenced in the figure) responsible of the sharp increase in the RMSD timeseries.

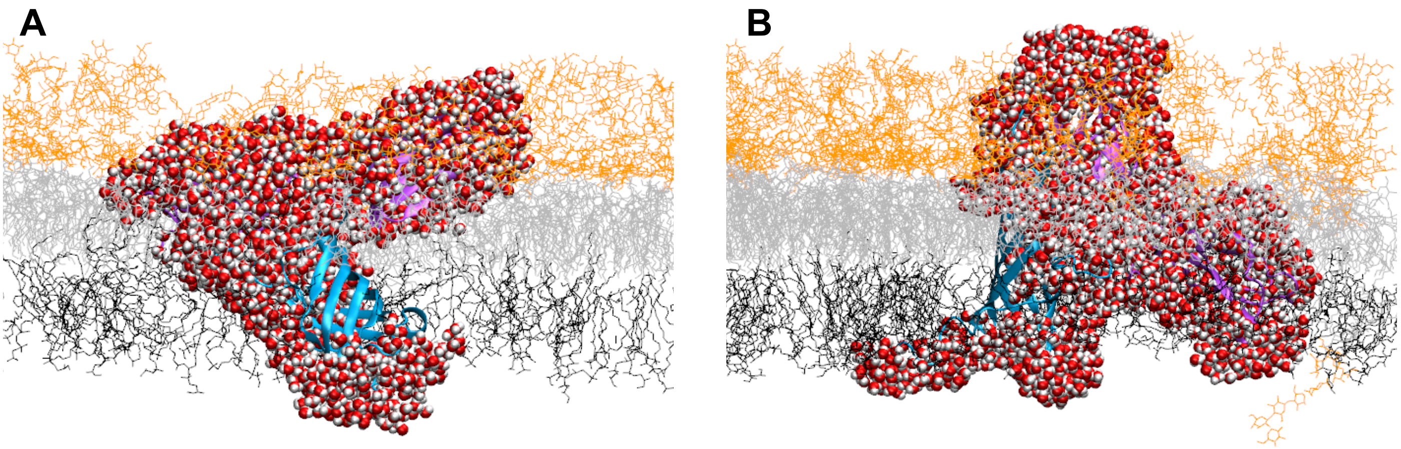

Figure S11. Examples of rejected structures due to membrane disruption and permeabilization. Lipid in gray and black, lipopolysaccharides in orange, OmpA in blue, two-unit nonRGD of fibronectin in purple. Red and white: water molecules.
